# Dyadic associations between patient self-efficacy, caregiver preparedness and self-care outcomes after stroke: a cross-sectional actor–partner interdependence model study

**DOI:** 10.1186/s12912-026-05226-2

**Published:** 2026-08-17

**Authors:** Ling Yuan, Chunyu He, Yuanmei Zhao, Yan Xie, Yurou Tian, Zhen Zhuo

**Affiliations:** 1 School of Nursing, Chengdu Medical College, Chengdu, Sichuan China; 2 The First Affiliated Hospital of Chengdu Medical College, Chengdu, Sichuan China

**Keywords:** Stroke, Self-care, Patient–caregiver dyads, Self-efficacy, Caregiver preparedness

## Abstract

**Background:**

Self-care after stroke develops within an interdependent patient–caregiver context. Although patient self-efficacy and caregiver preparedness are recognised as key determinants of post-stroke management, their dyadic associations with self-care-related variables remain insufficiently understood.

**Methods:**

A cross-sectional study was conducted among 198 patient–caregiver dyads recruited from three tertiary hospitals in China. Patient self-efficacy, caregiver preparedness, patient self-care ability and caregiver contribution were assessed using validated instruments. Dyadic associations were analysed using the actor–partner interdependence model within a distinguishable dyad regression framework. Standardised regression coefficients are reported.

**Results:**

Significant actor effects were identified for both members of the dyad. Patient self-efficacy was negatively associated with patient self-care ability (*β*= −0.242, *p* < 0.001), whereas caregiver preparedness was positively associated with caregiver contribution (*β* = 0.289, *p* < 0.001). No significant partner effects were observed. Patient self-efficacy and caregiver preparedness were positively correlated (*r* = 0.210, *p* < 0.01). The model explained 19.4% of the variance in patient self-care ability and 29.3% in caregiver contribution.

**Conclusions:**

In this sample, self-care ability and caregiver contribution were associated with individuals’ own psychological resources rather than cross-partner influences. Given the cross-sectional design and the absence of objective functional status measures, these findings should be interpreted as preliminary and hypothesis-generating rather than confirmatory. Intervention strategies should address patients and caregivers separately but concurrently, while recognising their distinct roles in self-care processes.

## Introduction

Stroke remains one of the leading causes of long-term disability worldwide and poses substantial challenges not only to people with stroke but also to their family caregivers. Beyond persistent physical impairments, stroke frequently disrupts cognitive and psychological functioning, resulting in prolonged dependence on ongoing care and rehabilitation after hospital discharge. In most cases, people with stroke must rely on sustained self-care and rehabilitation support to manage functional limitations and reduce the risk of secondary complications [1–3]. Within this context, family caregivers play a central role in assisting with daily activities, symptom monitoring and disease management, often becoming the primary source of long-term support [4–6]. Consequently, explanations of post-stroke recovery that focus solely on the individual may fail to capture the complexity of real-world care experiences. To better understand these dyadic dynamics, we review the key psychological determinants and the analytical framework in the following section.

## Background

Self-care is widely recognised as a cornerstone of chronic disease management. For individuals living with stroke, the capacity to engage in effective self-care is closely linked to functional recovery, complication prevention and improvements in quality of life. Previous studies have shown that active and sustained self-care behaviours are associated with better functional outcomes and fewer post-stroke complications [7–9]. However, self-care capacity among people with stroke is shaped by a range of factors, including disease severity, functional limitations and psychological resources [10]. Importantly, self-care behaviours do not occur in isolation. Rather, they are embedded within a dyadic context in which patients and caregivers interact continuously. Through shared daily routines, emotional exchanges and caregiving tasks, patients and caregivers influence each other’s behaviours and health outcomes, forming a highly interdependent relationship [11–13].

Self-efficacy has consistently been identified as a key psychological determinant of self-care behaviours. Higher levels of self-efficacy are generally associated with better adherence to rehabilitation, more consistent medication management and sustained engagement in health-promoting behaviours [14–17]. However, the direction and strength of this association may vary by disease stage and functional status, and some studies have reported non-linear or conditional relationships in acute illness contexts, warranting empirical examination in the present study. At the same time, caregiver preparedness for caregiving—reflecting psychological readiness, perceived competence and confidence in managing caregiving tasks—has been shown to influence both the quality of care provided and caregivers’ own physical and psychological well-being [18–21]. Caregivers who feel insufficiently prepared are more likely to experience caregiving burden and psychological distress, which may ultimately compromise the support they provide to people with stroke [22, 23].

Although the importance of patient self-efficacy and caregiver preparedness has been well documented, much of the existing literature has examined these factors from a single-person perspective. This approach overlooks the interdependence that characterises patient–caregiver relationships. The Actor–Partner Interdependence Model (APIM) offers a robust theoretical and analytical framework for dyadic research by allowing simultaneous estimation of actor effects (the influence of an individual’s characteristics on their own outcomes) and partner effects (the influence on their partner’s outcomes) [24–26]. APIM has been widely applied in studies of cancer, heart failure and dementia caregiving, yet its use in stroke patient–caregiver dyads remains limited [27–30].

Accordingly, the present study applied the APIM framework to examine the dyadic associations between patient self-efficacy, caregiver preparedness, and self-care-related variables in stroke patient–caregiver dyads. The primary objective was to investigate actor and partner effects of patient self-efficacy and caregiver preparedness on self-care-related outcomes. We hypothesised that: (1) patient self-efficacy would be associated with patient self-care ability, and caregiver preparedness would be associated with caregiver contribution (actor effects); and (2) patient self-efficacy would be associated with caregiver contribution, and caregiver preparedness would be associated with patient self-care ability (partner effects). A secondary objective was to examine the interdependence between patient self-efficacy and caregiver preparedness within stroke dyads.

## Methods

### Study design

A cross-sectional dyadic study design was employed to examine interdependence between people with stroke and their primary family caregivers. The study was conducted and reported in accordance with the Strengthening the Reporting of Observational Studies in Epidemiology (STROBE) guidelines for cross-sectional studies.

The Actor–Partner Interdependence Model (APIM) was used as the primary analytical framework. APIM is a statistical approach specifically designed for dyadic data that accounts for the non-independence of observations within dyads — a fundamental assumption that distinguishes dyadic analysis from conventional individual-level regression. The model simultaneously estimates two types of effects: actor effects, which refer to the influence of an individual’s predictor on their own outcome (e.g., a patient’s self-efficacy on their own self-care ability), and partner effects, which refer to the influence of an individual’s predictor on their partner’s outcome (e.g., a caregiver’s preparedness on the patient’s self-care ability). In addition, the model accounts for interdependence through two covariance parameters: the correlation between predictors within dyads and the residual correlation between the two outcome variables. This analytical approach permits a more complete understanding of how psychological resources operate within patient–caregiver relationships by acknowledging that patients and caregivers form an interdependent system rather than functioning as isolated individuals. All APIM analyses were conducted within a pooled ordinary least squares regression framework for distinguishable dyads, with role (patient vs. caregiver) as the distinguishing variable [31].

### Participants and setting

Participants were recruited using convenience sampling between March and July 2025 from the neurology and rehabilitation departments of three tertiary hospitals in Chengdu, China. people with stroke and their primary family caregivers were recruited as distinguishable dyads based on role (patient vs. caregiver).

### Sample size estimation

Sample size estimation was conducted using the APIMeM online power analysis tool for dyadic models [32]. Based on previous literature and theoretical assumptions, statistical power was set at 0.95. Expected effect sizes for both actor and partner effects were set at 0.20. The correlation between actor and partner predictors was assumed to be 0.60, and the residual correlation between outcome variables was set at 0.20. Under these assumptions, a minimum of 180 patient–caregiver dyads was required. Allowing for an estimated 10% rate of invalid questionnaires or attrition, at least 200 dyads were planned for recruitment. A total of 220 dyads were approached, and 198 complete dyads were included in the final analysis, yielding a valid response rate of 90.0%.

### Inclusion and exclusion criteria

Patients. Patients were eligible if they: (a) met the diagnostic criteria for stroke according to the Fourth National Conference on Cerebrovascular Diseases and had a diagnosis confirmed by CT or MRI; (b) were conscious and able to communicate effectively(determined by the attending physician and confirmed by research staff through brief verbal interaction); (c) were clinically stable and capable of completing the questionnaire independently(defined as no acute deterioration in vital signs or neurological status within the preceding 48 h, as assessed by the attending physician); and (d) provided written informed consent. Patients were excluded if they had severe comorbid cardiac, hepatic, pulmonary or renal disease, malignant tumours, or were participating in other research studies.

Caregivers. Caregivers were eligible if they: (a) were aged 18 years or older; (b) were identified as the primary caregiver defined as the family member who self-identified as providing the greatest amount of daily care to the patient, confirmed by both patient and caregiver report; (c) had adequate communication and comprehension abilities to complete the survey with guidance; and (d) provided written informed consent. Caregivers were excluded if they were paid professional caregivers, had severe health problems themselves, or were participating in other research studies.

### Measures

#### Sociodemographic and clinical information

A structured questionnaire was developed based on the study objectives and relevant literature. Patient information included demographic characteristics and clinical data, while caregiver information included demographic characteristics and caregiving-related variables.

#### Stroke self-care questionnaire

The self-care ability of people with stroke was assessed using the Stroke Self-Care Questionnaire developed by Wang Yanli and Wang Aiping [33].The scale consists of 33 items across four dimensions, including self-care concepts, self-care knowledge and self-care skills. Items are rated on a 5-point Likert scale, with higher scores indicating better self-care ability. The total score was calculated as the mean of all item scores.In the present study, the scale has demonstrated good internal consistency (Cronbach’s *α* = 0.915) and test–retest reliability (0.861).

#### Caregiver contribution to self-care of chronic illness inventory

Caregiver contribution to patients’self-care was measured using the Caregiver Contribution to Self-Care of Chronic Illness Inventory developed by Vellone et al. [34]. The instrument contains 19 items assessing contribution to self-care maintenance, monitoring and management, with higher scores indicating greater caregiver contribution. The Chinese version translated and validated by Chen et al. [35] has demonstrated good reliability (Cronbach’s α = 0.792–0.880) and validity. Items are rated on a 5-point Likert scale, with higher scores indicating greater caregiver contribution. Subscale scores and a total score can be calculated by averaging item responses.

#### Stroke Self-Efficacy Questionnaire (SSEQ)

Patient self-efficacy was measured using the Stroke Self-Efficacy Questionnaire developed by Jones et al. [36], which assesses confidence in performing daily activities and managing post-stroke challenges. The Chinese version of the Stroke Self-Efficacy Questionnaire (SSEQ), translated and validated by Yang Yujie and colleagues [37], was used to assess patient self-efficacy. The scale consists of 11 items, each scored on an 11-point scale ranging from 0 (not at all confident) to 10 (very confident). Total scores range from 0 to 110, with higher scores indicating greater self-efficacy. The Chinese version has demonstrated good reliability and validity in stroke populations. For ease of interpretation, item-level averages were used in descriptive analyses and correlation analyses, consistent with the scoring approach for the other instruments.

#### Caregiver Preparedness Scale (CPS)

Caregiver preparedness was assessed using the Caregiver Preparedness Scale developed by Archbold et al. [38], which evaluates caregivers’ perceived readiness to provide physical and psychological care and manage emergencies. The Chinese version translated by Liu Yanjin et al. [39] includes eight items evaluating perceived readiness to provide care. Items are rated on a 5-point Likert scale ranging from 1 (not at all prepared) to 5 (very well prepared), with higher scores indicating greater preparedness. The total score is calculated as the mean of all items. The scale has demonstrated excellent internal consistency (Cronbach’s *α* = 0.94) and test–retest reliability (0.92).

### Recruitment and data collection

Participant recruitment and data collection were conducted by trained research staff following a standardised protocol. Both inpatients and recently discharged people with stroke (defined as discharge within the preceding two weeks at the time of recruitment) attending follow-up visits, together with their primary caregivers, were eligible for inclusion. Patients and caregivers were screened according to the predefined inclusion and exclusion criteria.

Eligible patient–caregiver dyads were approached face to face, and the study aims, procedures and ethical considerations were explained in detail. Written informed consent was obtained from both patients and caregivers prior to participation. Clinical information, including stroke type, length of hospital stay and comorbid conditions, was extracted from hospital records. Sociodemographic data were collected through face-to-face interviews.

Study variables were assessed using structured questionnaires administered separately to patients and caregivers in the hospital setting. Questionnaires were completed independently whenever possible; when patients were unable to write due to physical limitations, caregivers recorded responses based on patients’ verbal answers. Research staff provided standardised instructions and technical assistance only, without offering any interpretative guidance. Completed questionnaires were checked on site for completeness, and missing or unclear responses were clarified immediately to ensure data quality.Questionnaires were completed between day 3 and day 7 of hospitalisation for inpatients, and within two weeks post-discharge for outpatients attending follow-up visits.

### Statistical analysis

Data were analysed using IBM SPSS Statistics version 26.0 (IBM Corp., Armonk, NY, USA) and Python version 3.11.0 with the statsmodels package (version 0.14.0). Descriptive statistics were used to summarise participant characteristics and study variables. Continuous variables are presented as means and standard deviations, and categorical variables as frequencies and percentages. Pearson correlation coefficients were calculated to examine bivariate associations.

Dyadic associations were analysed using the Actor–Partner Interdependence Model (APIM) within a pooled ordinary least squares regression framework for distinguishable dyads. Two regression equations were estimated simultaneously: (1) patient self-care ability as the outcome variable and (2) caregiver contribution to self-care as the outcome variable. Actor effects represent the association between an individual’s predictor and their own outcome, whereas partner effects represent the association between an individual’s predictor and their partner’s outcome.

To account for non-independence within dyads, correlations between predictors within dyads and residual correlations between outcome variables were modelled to account for interdependence into the analytical framework. All predictor variables were entered simultaneously. Regression coefficients are reported as standardised estimates (*β*).

Assumptions of linear regression were examined prior to model estimation. Linearity, homoscedasticity and normality of residuals were assessed using diagnostic plots.Normality of continuous variables was formally tested using the Shapiro–Wilk test. Multicollinearity was evaluated using variance inflation factors (VIF), with all VIF values below 5 indicating no substantial multicollinearity.

Statistical significance was set at *p* < 0.05 (two-tailed). Model explanatory power was evaluated using *R²*values for each outcome variable.

Missing data were handled using complete-case analysis. Only dyads providing complete data on key study variables were included in the final analyses.

### Ethical considerations

The study protocol was reviewed and approved by the Ethics Committee of Chengdu Medical College (Approval No. CY2024NO.103). All procedures were conducted in accordance with the Declaration of Helsinki. Written informed consent was obtained from all participants prior to data collection.

## Results

### Participant characteristics

A total of 396 valid questionnaires were collected, corresponding to 198 patient–caregiver dyads (each dyad comprising one patient and one primary caregiver). After data completeness checks and screening for missing values, all 198 dyads were included in the final analyses. Sociodemographic and clinical characteristics of patients and caregivers are presented in Tables 1 and 2.

The mean age of patients was 69.29 years (SD = 12.99), and most were male (57.1%). More than half of the patients had an educational level of primary school or below (57.6%), were married (80.8%), and lived in townships (37.9%) or rural areas (33.8%). Ischaemic stroke (38.4%) and haemorrhagic stroke (60.1%) were the most common stroke types. The mean length of hospital stay was 8.03 days (SD = 7.39), which approximated a normal distribution based on the Shapiro–Wilk test (W = 0.985, *p* = 0.062). This metric served as a proxy for disease stage, as the study did not systematically distinguish between inpatients and post-discharge outpatients. Most patients had at least one comorbid chronic condition (91.4%).

Caregivers had a mean age of 51.87 years (*SD* = 13.21), and the majority were female (56.6%). Most caregivers were adult children (54.0%) or spouses (33.8%) of the patients, and more than half lived with the patient (58.6%). Approximately two-thirds of caregivers had an educational level of junior high school or below (63.1%). The mean daily caregiving time was 7.70 h (SD = 3.93).

Regarding the main study variables, mean scores for patient self-efficacy(calculated as the item average of the SSEQ, ranging from 0 to 10, with higher scores indicating greater confidence) and patient self-care ability were 3.67 (SD = 0.72) and 3.54 (SD = 0.68), respectively, indicating moderate-to-high levels. Mean scores for caregiver preparedness and caregiver contribution were 3.21 (SD = 0.74) and 3.09 (SD = 0.69), reflecting moderate levels overall.

**Table 1:** Demographic and clinical characteristics of patients (*N* = 198)

Variable	Category	*n* (%) or Mean ± SD
Age (years)	—	69.29 ± 12.99
Sex	Male	113(57.1%)
	Female	85(42.9%)
Education level	Primary school or below	114(57.6%)
	Junior high school	55(27.8%)
	Senior high school / Technical secondary school	20(10.1%)
	College or above	9(4.5%)
Marital status	Married	160(80.8%)
	Unmarried	38(19.2%)
Residence	Urban	56(28.3%)
	Town	75(37.9%)
	Rural	67(33.8%)
Stroke type	Ischemic	76(38.4%)
	Hemorrhagic	119(60.1%)
Length of hospital stay (days)	—	8.03 ± 7.39
Number of comorbid chronic diseases	None	17(8.6%)
	≥ 1	181(91.4%)

Note: Continuous variables are presented as mean ± standard deviation, and categorical variables are presented as number (percentage)

**Table 2:** Demographic and caregiving characteristics of caregivers (*N* = 198)

Variable	Category	*n* (%) or Mean ± SD
Age (years)	—	51.87 ± 13.21
Sex	Male	86(43.4%)
	Female	112(56.6%)
Relationship to patient	Spouse	67(33.8%)
	Child	107(54.0%)
	Other	24(12.2%)
Education level	Junior high school or below	125(63.1%)
	Senior high school or above	73(36.9%)
Living with patient	Yes	116(58.6%)
	No	82(41.4%)
Daily caregiving time (hours/day)	—	7.70 ± 3.93

Note: Continuous variables are presented as mean ± standard deviation, and categorical variables are presented as number (percentage)

### Descriptive statistics and correlation analysis

Descriptive statistics for the main study variables are shown in Table 3. Patient self-efficacy and self-care ability were at moderate to relatively high levels, whereas caregiverpreparedness and caregiver contribution were within the moderate range.

**Table 3:** Descriptive statistics of study variables (*N* = 198 dyads)

Variable	*N*	Minimum	Maximum	Mean ± SD
Patient self-efficacy	198	1.25	4.88	3.67 ± 0.72
Patient self-care ability	198	1.10	4.95	3.54 ± 0.68
Caregiver preparedness	198	1.30	4.70	3.21 ± 0.74
Caregiver contribution	198	1.05	4.85	3.09 ± 0.69

Note: Scores for all variables are standardized or averaged scale scores; higher scores indicate higher levels of the corresponding ability or capacity

Correlation analyses among study variables within patient–caregiver dyads are presented in Table 4. A significant positive correlation was observed between patient self-efficacy and caregiver preparedness (*r* = 0.210, *p* < 0.01), indicating a degree of association between psychological resources within the dyad. In contrast, patient self-care ability was negatively correlated with caregiver contribution; however, this association did not reach statistical significance (*r* = − 0.098, *p* > 0.05).

**Table 4:** Correlation analysis of study variables (*N* = 198 dyads)

Variable	Patient Self-Efficacy	Caregiver Preparedness	Patient Self-Care Ability	Caregiver Contribution
Patient Self-Efficacy	1	0.210**	—	—
Caregiver Preparedness	0.210**	1	—	—
Patient Self-Care Ability	—	—	1	-0.098
Caregiver Contribution	—	—	−0.098	1

Note: *p* < 0.01, *N* = 198 dyads

### Actor–Partner Interdependence Model analysis

**Fig. 1:**
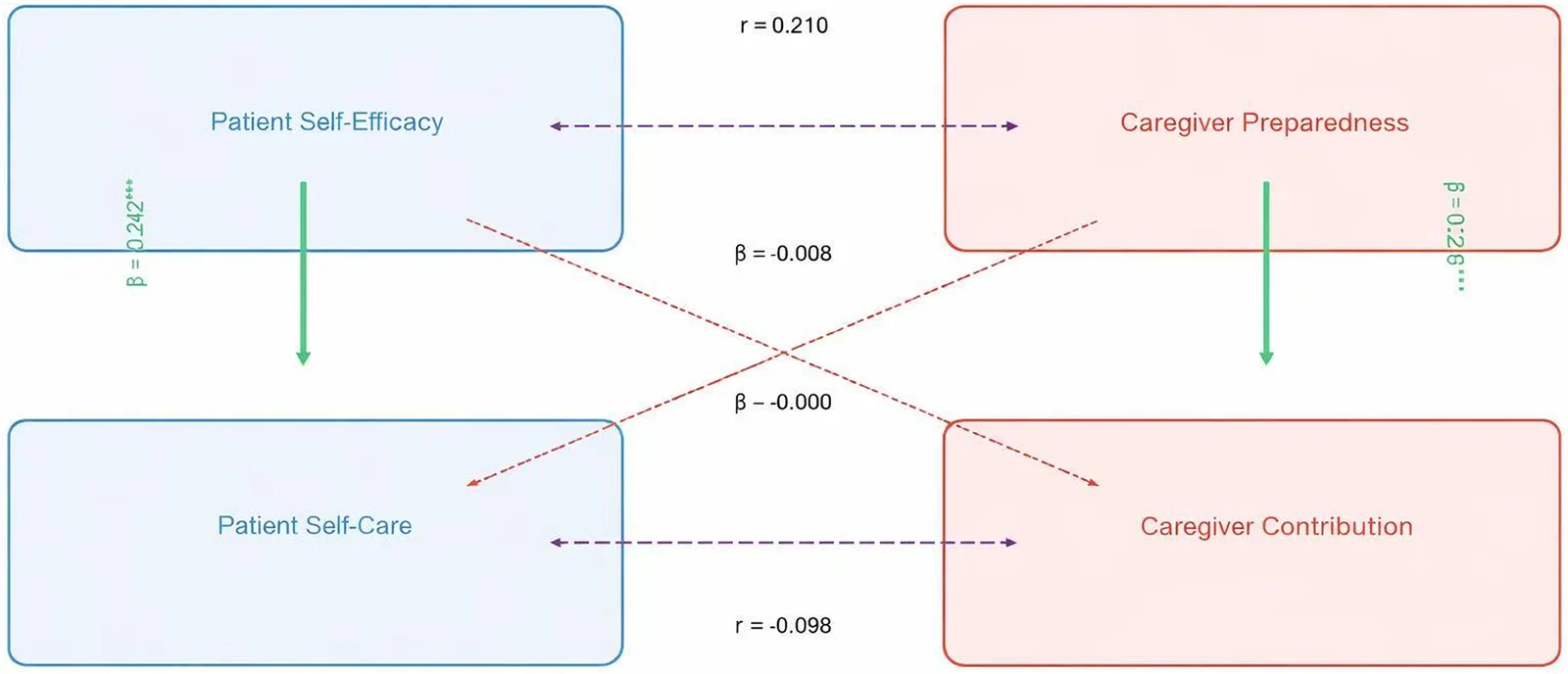
APIM analysis results (Based on empirical data). Note: Solid lines =actor effects, dashed lines = partner effects, double-headed lines

The structure of the Actor–Partner Interdependence Model (APIM) is illustrated in Fig. 1, and the regression results are summarised in Table 5. Path coefficients reported in Table 5 correspond to the paths shown in Fig. 1.

**Table 5:** Actor–Partner interdependence model (APIM) path regression results (*N* = 198 dyads)

Path Type	Specific Path	β	SE	t	*p*	Significance
Actor effect	Patient self-efficacy → Patient self-care	−0.242	0.036	−6.731	< 0.001	***
Actor effect	Caregiver preparedness → Caregiver contribution	0.289	0.032	8.908	< 0.001	***
Partner effect	Caregiver preparedness → Patient self-care	−0.000	0.054	−0.005	0.996	n.s.
Partner effect	Patient self-efficacy → Caregiver contribution	−0.032	0.039	−0.809	0.420	n.s.

Note: β = standardized regression coefficient; n.s. = not significant; *** *p* < 0.001

#### Actor effects

Significant actor effects were observed for both patients and caregivers. Patient self-efficacy was a significant negative predictor of patient self-care ability (*β* = −0.242, SE = 0.036, *t* = − 6.731, *p* < 0.001). In addition, caregiver preparedness showed a significant positive actor effect on caregiver contribution to self-care (*β* = 0.289, SE = 0.032, *t* = 8.908, *p* < 0.001).

#### Partner effects

No significant partner effects were identified. Caregiver preparedness did not significantly predict patient self-care ability (*β* = −0.000, SE = 0.054, *t* = − 0.005, *p* = 0.996), and patient self-efficacy was not a significant predictor of caregiver contribution (*β* = −0.032, *SE* = 0.039, *t* = − 0.809, *p* = 0.420).

### Covariances and explained variance

Covariance analyses indicated a significant positive correlation between patient self-efficacy and caregiver preparedness (*r* = 0.210, *p* < 0.01), suggesting interdependence in psychological resources within the dyad. The residual correlation between patient self-care ability and caregiver contribution was weak and negative (*r* = − 0.098).

Regarding model explanatory power, the APIM accounted for 19.4% of the variance in patient self-care ability and 29.3% of the variance in caregiver contribution (Table 6). *R² *values ranged from 0.194 to 0.293, indicating moderate explanatory power.

**Table 6:** APIM model fit and explained variance

Outcome Variabl	*R*²
Patient self-care	0.194
Caregiver contribution	0.293

Note: R² indicates the proportion of variance in the outcome variable explained by the model

## Discussion

This study provides novel dyadic evidence regarding the psychological determinants of self-care among stroke patient–caregiver dyads. Significant actor effects were identified for both patients and caregivers, whereas partner effects were not statistically significant. These findings suggest that, within this sample, self-care outcomes were more strongly associated with individuals’ own psychological resources than with direct cross-partner influences. The results underscore the importance of considering both members of the dyad while recognising the potentially distinct pathways through which psychological factors operate [11, 40].

### Interpretation of the negative actor effect

An unexpected yet statistically robust finding was the negative association between patient self-efficacy and patient self-care ability. Classical self-efficacy theory posits that greater perceived capability facilitates behavioural engagement [14, 30]. Consistent with this theoretical framework, previous studies in stroke populations have generally reported positive associations between self-efficacy and functional recovery or self-management outcomes [41, 42]. However, this assumption may not fully apply in acute or early post-stroke rehabilitation contexts [3, 27].

To ensure the robustness of this unexpected finding, we first examined whether statistical errors or measurement issues could have contributed to the result. We carefully reviewed our data entry and scoring procedures and confirmed that all items in both the Stroke Self-Efficacy Questionnaire (SSEQ) and the Stroke Self-Care Questionnaire (SSCQ) were scored in the correct direction, with no reverse-scoring errors. The negative association remained consistent across the main APIM analysis, suggesting that it was unlikely to be an artefact of simple data errors or scoring mistakes. However, given the cross-sectional design and the absence of objective functional status measures, we cannot rule out the possibility that unmeasured confounders — particularly physical functioning and stroke severity — may have influenced the observed association. We therefore interpret this finding as preliminary and hypothesis-generating, requiring confirmation in future longitudinal studies with comprehensive functional assessments.

First, self-efficacy measured during hospitalisation may reflect psychological optimism or coping orientation rather than objectively grounded functional competence. Patients with relatively limited physical function may report high confidence as a motivational or adaptive response [27], resulting in a discrepancy between perceived capability and actual performance. Second, in early recovery stages, intensive caregiver involvement may reduce patients’ opportunities for independent self-care behaviours, thereby attenuating the behavioural manifestation of self-efficacy [4, 5]. Third, cultural norms emphasising family responsibility in caregiving may further shape this dynamic, particularly in contexts where family members assume substantial caregiving roles during hospitalisation [24, 25].

Therefore, rather than contradicting self-efficacy theory, this finding may reflect stage-specific and context-dependent dynamics in stroke rehabilitation. It highlights the need to interpret self-efficacy alongside objective functional status and environmental support structures.

### Absence of partner effects in early rehabilitation

No significant partner effects were observed in this study: caregiver preparedness was not directly associated with patient self-care ability, and patient self-efficacy was not directly associated with caregiver contribution. Within the Actor–Partner Interdependence Model (APIM), actor and partner effects represent distinct pathways of influence, and the relative strength of these pathways may vary depending on outcome type and contextual factors [40, 41]. The present findings suggest that, in this sample, self-care outcomes were more strongly associated with individuals’ own characteristics than with direct cross-partner influences.

Importantly, this finding does not imply that dyadic interaction is unimportant. Theoretical frameworks such as the Theory of Dyadic Illness Management emphasise interdependence in chronic illness contexts [11], and meta-analytic evidence supports the existence of partner effects across various health outcomes [43]. Rather, our results indicate that individual-level pathways may predominate for these specific behavioural outcomes and at this early stage of stroke recovery.

One possible explanation is that patient self-care behaviours are highly dependent on personal psychological resources, functional status and illness-related knowledge, which may limit the extent to which caregiver preparedness alone can directly influence patient behaviour. Similarly, caregiver contribution may be primarily shaped by caregivers’ own preparedness, role perception and available resources. Previous dyadic studies in stroke and chronic illness contexts have also reported stronger actor effects, particularly when outcomes reflect individual behavioural engagement or role-specific effort [26, 40, 41, 44]. For example, Pucciarelli et al. [44] found significant actor effects but no partner effects of caregiver preparedness on stroke patient outcomes in an Italian sample, consistent with our findings.Similarly, Petrizzo et al. [45] demonstrated that caregiver preparedness moderated the relationship between depression and stroke-specific quality of life in a longitudinal stroke dyad sample. However, some studies in other chronic illness populations — particularly in heart failure and cancer — have detected significant partner effects [29, 43], suggesting that the presence and strength of partner effects may vary by illness type, outcome measure, and recovery stage. These comparisons underscore the need for further research to clarify the conditions under which partner effects emerge in stroke populations.

In addition, most participants in this study were hospitalised or recently discharged. During early recovery, dyadic interaction patterns may not yet be fully stabilised, and partner effects may emerge later or through more complex mediated pathways. Longitudinal research is needed to examine whether cross-partner influences become more prominent as recovery progresses [26, 44].

### Dyadic interdependence of psychological resources

Despite the absence of direct partner effects on behavioural outcomes, a significant positive association was observed between patient self-efficacy and caregiver preparedness. This finding supports the assumption that patients and caregivers form an interdependent system, as proposed in dyadic illness management and couples coping frameworks [11, 12]. These models suggest that psychological processes within dyads are interconnected and mutually shaped over time. Empirical evidence from dyadic coping research further indicates that emotional and cognitive resources often co-evolve within close relational contexts [43].

Shared experiences of illness, rehabilitation and caregiving may therefore contribute to convergence in confidence, expectations and coping orientations, even when behavioural outcomes remain primarily individually driven. Longitudinal stroke research has similarly demonstrated parallel adaptation processes within patient–caregiver dyads during recovery [26, 44].

In contrast, the residual association between patient self-care ability and caregiver contribution was weak after accounting for key predictors, further suggesting that behavioural outcomes were largely associated with individual-level factors in this context.

### Implications for nursing practice

These findings have important implications for nursing practice in stroke rehabilitation. First, systematic assessment of both patient self-efficacy and caregiver preparedness should be integrated into routine clinical evaluation, as each appears to independently influence self-care–related outcomes. Theoretical and empirical research has consistently demonstrated that self-efficacy plays a central role in promoting health-related behaviour change [14, 30], and structured self-management interventions have shown beneficial effects in stroke populations [16].

Second, interventions aimed at enhancing patient self-efficacy should be aligned with patients’ functional capacity and opportunities for behavioural engagement, ensuring that confidence is supported by skill development and graded task participation. This approach is consistent with self-efficacy theory, which emphasises mastery experience and guided practice as key mechanisms of behavioural change [14].Specifically, rehabilitation nurses could: (a) assess patients’ baseline functional status and set incremental, achievable self-care goals (e.g., progressing from assisted to independent grooming); (b) provide structured opportunities for practising self-care tasks during hospitalisation with real-time feedback; and (c) use verbal persuasion and vicarious learning (e.g., peer modelling) to reinforce confidence, while carefully monitoring for discrepancies between perceived and actual capability that may indicate overconfidence.

Third, structured caregiver preparation programmes delivered by nursing professionals during hospitalisation and discharge planning may strengthen caregivers’ active contribution to patient self-care. Previous studies have shown that caregiver readiness and preparedness are associated with caregiving outcomes and patient adjustment [20, 21]. Such programmes may include skills training, role clarification and tailored education to enhance caregiving confidence. To enhance practical operability, these programmes could be delivered through: (a) brief bedside coaching sessions during hospitalisation (e.g., 15–20 min daily) focused on specific caregiving tasks such as positioning, transferring, and medication management; (b) structured discharge planning meetings that clarify role expectations and provide written care guides; and (c) telephone follow-up within 48 h post-discharge to address emerging concerns and reinforce preparedness.

Overall, adopting a dyadic yet role-sensitive approach, as recommended in dyadic illness management frameworks [11], may better reflect the complexity of stroke recovery and support sustainable self-care practices after discharge.

### Limitations and future research

Several limitations should be acknowledged. First, the cross-sectional design precludes causal inference. Although the Actor–Partner Interdependence Model allows examination of dyadic associations, it does not establish temporal directionality, particularly in cross-sectional applications [22, 23]. Longitudinal studies are needed to examine how actor and partner effects evolve across different stages of stroke recovery. In addition, although length of hospital stay was reported as a proxy for disease stage, the study did not systematically distinguish between inpatients and post-discharge outpatients. This may have obscured stage-specific patterns, as patients at different recovery phases may differ in self-care ability and caregiving needs. Future studies should explicitly stratify or adjust for recruitment setting (inpatient vs. outpatient) to address this limitation.

Second, and most importantly, objective measures of functional status (e.g., Barthel Index, modified Rankin Scale, or NIH Stroke Scale) were not included in the APIM models. This is a major limitation of the present study, as self-care ability in stroke survivors is strongly influenced by physical functioning and stroke severity. Without such data, our interpretations of the negative association between patient self-efficacy and self-care ability remain speculative and should be viewed as hypothesis-generating rather than confirmatory. Future research incorporating standardised functional assessments is needed to determine whether this finding reflects stage-specific psychological dynamics, functional constraints, or a combination of both.

Finally, potential mediators and moderators, such as social support, caregiving duration and rehabilitation intensity, were not examined. Incorporating these variables in future longitudinal dyadic studies may provide a more comprehensive understanding of patient–caregiver interdependence in stroke rehabilitation, as suggested by previous dyadic longitudinal research [44].

## Conclusion

Using the Actor–Partner Interdependence Model (APIM), this study systematically examined the associations between patient self-efficacy, caregiver preparedness, and self-care–related variables within stroke patient–caregiver dyads during hospitalisation and early post-discharge. Significant actor effects were observed for both patients and caregivers, whereas no significant partner effects were identified. Within this sample, and with the caveat that objective functional status measures were not included, self-care ability and caregiver contribution were associated with individuals’ own psychological resources rather than cross-partner influences. However, given the cross-sectional design and the absence of functional status measures, these findings should be interpreted as preliminary and hypothesis-generating rather than confirmatory.

Higher patient self-efficacy was negatively associated with self-care ability, suggesting stage-specific dynamics in early rehabilitation, whereas caregiver preparedness showed a positive association with caregiver contribution. Psychological resources — patients’ self-efficacy and caregivers’ perceived preparedness — were interdependent within dyads.

Overall, these findings support the value of a dyadic analytical perspective in stroke care research. Given the absence of partner effects, intervention strategies should address patients and caregivers separately but concurrently — enhancing patient self-efficacy in alignment with functional capacity, while strengthening caregiver preparedness through structured training and education. Future longitudinal research incorporating objective functional assessments is needed to validate these findings and examine whether cross-partner influences emerge at later stages of recovery.

## Data Availability

The datasets generated and/or analysed during the current study are available from the corresponding author on reasonable request.

## Abbreviations

APIM: Actor–Partner Interdependence Model
CC-SC-CII: Caregiver Contribution to Self-Care of Chronic Illness Inventory
CPS: Caregiver Preparedness Scale
CI: Confidence Interval
SD: Standard Deviation
SSCQ: Stroke Self-Care Questionnaire
SSEQ: Stroke Self-Efficacy Questionnaire
